# Cannabinoids in Periodontology: Where Are We Now?

**DOI:** 10.3390/antibiotics12121687

**Published:** 2023-11-30

**Authors:** Yésica Carmona Rendón, Hernán Santiago Garzón, Bruno Bueno-Silva, Roger M. Arce, Lina Janeth Suárez

**Affiliations:** 1 Departamento de Ciencias Básicas y Medicina Oral, Facultad de Odontología, Universidad Nacional de Colombia, Bogotá 111321, Colombia; ycarmona@unal.edu.co; 2 Programa de Doctorado en Ingeniería, Facultad de Ingeniería, Pontificia Universidad Javeriana, Bogotá 110231, Colombia; garzonhernan@javeriana.edu.co; 3 Departamento de Biociências, Faculdade de Odontologia de Piracicaba, Universidade de Campinas (UNICAMP), Piracicaba 13414-903, Brazil; brunobue@unicamp.br; 4 Department of Periodontics and Oral Hygiene, University of Texas School of Dentistry at Houston, Houston, TX 77054, USA; roger.m.arce@uth.tmc.edu; 5 Centro de Investigaciones Odontológicas, Facultad de Odontología, Pontificia Universidad Javeriana, Bogotá 110231, Colombia

**Keywords:** cannabis, cannabinoids, periodontitis, regeneration

## Abstract

Introduction: Cannabinoids are a well-documented treatment modality for various immune and inflammatory diseases, including asthma, chronic obstructive pulmonary disease, Crohn’s disease, arthritis, multiple sclerosis, and a range of neurodegenerative conditions. However, limited information is available regarding the therapeutic potential of cannabinoids in treating periodontal disease. Objective: The objective of this study is to analyze the current evidence on the antibacterial and immunomodulatory effects of cannabis and its role in the healing and regeneration processes within periodontal tissues. Results: This review discusses the potential role of cannabinoids in restoring periodontal tissue homeostasis. Conclusions: The examination of the endocannabinoid system and the physiological effects of cannabinoids in the periodontium suggests that they possess immunomodulatory and antibacterial properties, which could potentially promote proper tissue healing and regeneration.

## 1. Introduction

The clinical use of cannabis as a therapeutic approach for multiple diseases has increased in the last decade due to the approval of its medical use in the legislation of multiple countries. In the United States, 38 states, three territories, and the District of Columbia have legalized the medical use of cannabis derivatives. At least eight countries in Latin America (Argentina, Brazil, Chile, Colombia, Mexico, Paraguay, Peru, and Uruguay) and countries in Europe, such as Austria, Belgium, Croatia, Cyprus, France, and Germany, have legalized its medical use. The United Kingdom and Spain also have some cannabinoid-based medications available for prescription. Outside Europe, Australia has implemented a medical cannabis program.

Historically, publications on cannabis have primarily focused on its psychoactive effects and their side effects on various organs, including the oral cavity and its different niches [1,2]. However, there is currently a growing body of evidence regarding the safety and effectiveness of cannabinoids with potential therapeutic uses in diseases of different natures, including infectious and inflammatory conditions [3,4,5,6,7].

Periodontitis is an inflammatory condition affecting the supporting tissues of the teeth, often associated with microbial dysbiosis and systemic implications that play a crucial role in its connection to multiple systemic diseases [8,9,10,11,12]. The condition is characterized by a bidirectional imbalance between the microorganisms in the periodontal environment and the host’s immune response, which mediates the tissue loss typical of periodontal diseases [13]. Therefore, using antimicrobials and immunomodulators to treat periodontal diseases emerges as a desirable adjunct therapeutic modality to reduce periodontal damage and promote tissue regeneration.

For over a decade, researchers have extensively examined the effects of cannabis on the regulation of inflammation through the CB2 receptor (a non-psychoactive cannabinoid receptor) [14]. Recently, the mechanisms of action associated with cannabis have been proposed to be beneficial in addressing a range of inflammatory clinical conditions; these conditions include motor disorders [15,16], as well as other neurological and neurodegenerative diseases such as Alzheimer’s [17] and epilepsy [18]. Additionally, cannabis has shown promise in addressing conditions like inflammatory bowel disease [19], inflammatory and neoplastic skin diseases [20], and even in preventing the formation of fibrosis [21,22].

The literature’s interest in the antimicrobial properties of cannabis bioactive compounds has grown in the last decade. Recent research indicates that these compounds have effects at low concentrations, typically ranging from 1 to 64 ug/mL, depending on the specific strain. The primary compounds derived from *Cannabis sativa* (*C. sativa*) that have been tested include its essential oil, cannabidiol (CBD), cannabidiolic acid (CBDA), cannabigerol (CBG), cannabidivarin (CBDV), and *C. sativa* water extract [23]. In the case of Gram-positive bacteria, such as *Staphylococcus aureus*, it has been suggested that CBD’s antimicrobial mechanism of action involves reducing the peptidoglycan content of the bacterial cell wall and increasing bacterial membrane permeability [24].

Therefore, this comprehensive review aims to analyze the current evidence available on the antibacterial and immunomodulatory capabilities of cannabis and its derivatives against periodontopathic bacteria. For this purpose, this review is divided into three parts: first, it explores the generalities of cannabinoids and their receptors and the biological basis for their use in inflammatory/infectious diseases, given their effect on inflammation and the microbiota. Second, the review summarizes evidence on the antibacterial/immunomodulatory properties concerning the pathogenesis of periodontitis. Lastly, the review delves into a detailed examination of the existing literature on the impact of cannabis on the biology of periodontium and its potential therapeutic applications.

## 2. Cannabinoids Definitions

A cannabinoid is defined as any chemical substance that can bind to cannabinoid receptors in the human body, whether produced by the body’s cells, of synthetic origin, or derived from the Cannabis sativa plant. They all have in common the capacity to produce effects like those generated by the plant [25]. Cannabis contains three different bioactive molecules: flavonoids, terpenoids, and cannabinoids [26]. Phytochemical cannabinoids are secondary metabolites produced naturally. They include some psychoactive compounds, such as Δ9-tetrahydrocannabinol (Δ9-THC), and several non-psychoactive compounds, including cannabidiol (CBD), cannabigerol (CBG), cannabinol (CBN), and cannabichromene (CBC) [27,28]. In addition to phytocannabinoids, cannabinoids can fall into two categories: endocannabinoids (produced in the body) and synthetic cannabinoids (artificial chemical compounds). Most of the endogenous cannabinoids identified so far act as cannabinoid receptor agonists, with varying levels of efficacy, either high or low [29].

Cannabinoid receptor agonists can also be categorized based on differences in their chemical structure, falling into two main groups: classical and non-classical compounds, including aminoalkylindoles and eicosanoids [29,30]. The classical group comprises phytocannabinoids such as Δ9-THC, cannabinol, and synthetic analogs. The eicosanoid group primarily consists of endocannabinoids naturally produced within the body, including compounds like anandamide (arachidonoyl ethanolamide-AEA), virodhamine (O-arachidonoyl ethanolamine), and 2-arachidonoylglycerol (2-AG), along with several synthetic analogs of anandamide. The non-classical and aminoalkylindole groups consist of synthetic cannabinoids [31].

**Figure 1:**
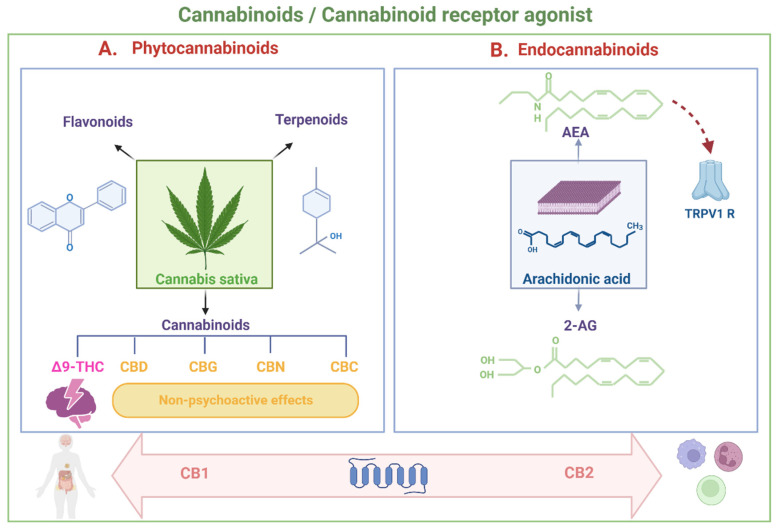
Cannabinoids derived from the cannabis sativa plant are known as phytocannabinoids, including the psychoactive component Δ9-tetrahydrocannabinol (Δ9-THC), cannabidiol (CBD), cannabigerol (CBG), cannabinol (CBN), and cannabichromene (CBC), which lack such effects. Flavonoids and terpenoids constitute other bioactive molecules contained in the plant; terpenoids are secondary metabolites with organoleptic properties that give them differential properties (**A**). Endocannabinoids (compounds produced naturally by the human body) belong to the eicosanoid group, and its two main representatives are anandamide (arachidonoyl ethanolamide-AEA), and 2-arachidonoylglycerol (2-AG), which act as cannabinoid receptor agonists. Both phytocannabinoids and endocannabinoids act on the cannabinoid receptors CB1 and CB2. CB1 receptors are widely distributed in the human body, with high expression in the central nervous system, while CB2 receptors are mainly found in the cells of the immune system (**B**).

The most extensively studied endogenous cannabinoids are AEA and 2-AG. These two compounds are produced as needed and are removed from their action sites through cellular uptake processes. AEA and 2-AG, the active enzymes responsible for their synthesis and degradation, and the cannabinoid receptors CB1 and CB2 collectively constitute the endocannabinoid system. This system is recognized as a crucial signaling system with multiple functions in the human body. It plays a vital role in the central and peripheral control of intestinal function [32], regulates brain homeostasis by modulating the hypothalamic–pituitary–adrenocortical (HPA) axis [33], and facilitates the interaction and function coordination between the immune, endocrine, and nervous systems [34], among other functions (Figure 1A,B). 

The discovery of the endocannabinoid system has extended beyond cannabis research. It has sparked interest in the pharmacology of phytocannabinoids and their synthetic analogs and in understanding the physiological and pathological events they might initiate.

## 3. Cannabinoid Receptors

The physiological impacts of cannabinoids are facilitated through their interaction with cannabinoid receptors [31,35], encompassing CB1 and CB2. These receptors are membrane proteins, existing as single polypeptides characterized by seven transmembrane α-helices. They operate as G-protein-coupled receptors, inhibiting adenylate cyclase and calcium channels while concurrently inducing the opposite effect on potassium channels [26,29,36,37,38,39,40,41].

Cannabinoid receptors exhibit a widespread distribution throughout the body, yet their expression varies. CB1 receptors are commonly located in the central nervous system, specifically in neuronal terminals within the basal ganglia, cerebellum, hippocampus, neocortex, hypothalamus, and limbic cortex. On the other hand, CB2 receptors are predominantly expressed in immune system cells [31,36,37,40,41,42], actively participating in the modulation of cytokine release and cell migration [26,29,38]. The cell types expressing these receptors encompass phagocytic cells such as neutrophils and macrophages and B and T lymphocytes, monocytes, and mastoid cells [26,29,31,36,37,39,40,41,42,43].

Endocannabinoids not only regulate the activity of cannabinoid receptors but also modulate cellular homeostasis through interactions with various targets. This includes a degree of “promiscuity”, where they can activate or inhibit different molecular targets, ion channels, and nuclear receptors. For instance, anandamide has demonstrated its action on the non-cannabinoid receptor TRPV1 (transient receptor potential vanilloid type 1 channel [40].

Immune cells express both CB1 and CB2 receptors, with a predominance for CB2 [29,44]. The expression of these receptors depends on the immune cell’s stimulation and activation state, with higher expression found in descending order on B and T lymphocytes, NK cells, monocytes, neutrophils, CD8 leukocytes, and CD4 leukocytes [45]. Furthermore, recent research has suggested that the endocannabinoid system interacts with the cellular signaling system of reactive oxygen species, modulating its function [46].

## 4. Cannabinoid Effects on the Immune System

Cannabinoids, both endocannabinoids and exo-cannabinoids, influence various facets of the immune system’s effector function, encompassing cytokine release, cell proliferation, and effector enzyme levels [31,36,45,47].

Cannabinoids have been observed to decrease the release of pro-inflammatory cytokines such as IL1, IL-12, IL-2, IL-6, tumor necrosis factor α(TNF-α), and interferon-gamma (IFN-γ) while increasing the production of anti-inflammatory cytokines associated with the control of the inflammatory process, including IL-4 and IL-10 [36,37]. Additionally, cannabinoids can reduce prostaglandin E2 and tissue cyclooxygenase (COX) activity and suppress serum immunoglobulin levels [37]. 

CBD has shown the ability to inhibit the migration of neutrophils and modulate the expression of TNF-α and IL-1β in experimental animal models of various inflammatory conditions, including neuronal and renal inflammation, inflammatory bowel disease, lung lesions, and asthma [48,49,50,51,52]. CBD and THC have the capability to activate immunoregulatory genes in both the mitogen-activated protein kinases (MAPK) and Janus kinases (JAKs)/signal transducer and activator of transcription proteins (STATs) families. Additionally, CBD can induce negative regulators of nuclear factor kappa-light-chain-enhancer of activated B cells (NF-κB) and activator protein 1 (AP-1), thereby emphasizing its immunomodulatory properties [53]). 

Similarly, CBD has been shown to affect the JAK/STAT pathway, reducing the production of IL-2 and IFN-γ in mouse splenocytes and inhibiting interleukin-10 (IL-10) in human leukemic T cells, demonstrating its potential for immunosuppression. Moreover, CBD has been found to reduce pro-inflammatory signaling by modulating the IFNβ/STAT pathway, decreasing the expression of IL-1β, IL-6, and IFNβ in LPS-activated microglial cells. This effect is achieved by suppressing NF-κB-mediated transcription and enhancing the anti-inflammatory phosphorylation of the signal transducer and activator of transcription 3 (STAT3) while reducing the pro-inflammatory phosphorylation of STAT1 [54].

CBG exhibits anti-inflammatory effects by reducing nitric oxide production in macrophages through CB2 receptor activation. It also inhibits T cell proliferation and Th1/Th17 cytokine secretion, modulates macrophage polarization to an anti-inflammatory profile, and ameliorates the severity of experimental autoimmune encephalomyelitis (EAE) in mice by activating CB2 and peroxisome proliferator-activated receptor gamma (PPARγ) receptors [55].

**Figure 2:**
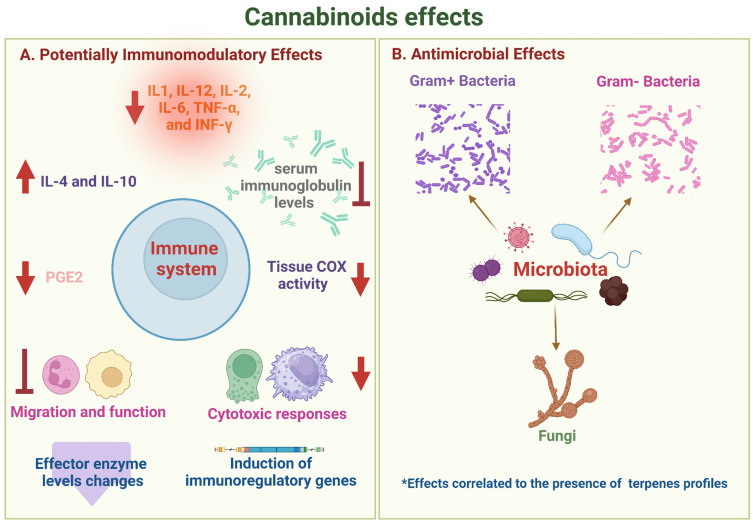
Both endocannabinoids and exo-cannabinoids influence various facets of the immune system. Effects on effector cells of both innate immunity (PMNn, macrophages, and NK cells) and acquired immunity (CD4+ and CD8+ T cells, and B lymphocytes) have been described, influencing cell proliferation and the release of pro-inflammatory cytokines (IL-12, IL-6, TNFa, and IFNg) and regulators (IL.10 and TGFb); the levels of effector enzymes are also affected (**A**). In relation to the effects on the microbiota, cannabinoids have shown antibacterial activity both in planktonic Gram+ and Gram- bacteria and in preventing the formation of certain biofilms, in addition to clear fungicide activity (**B**). * Antimicrobial effects of cannabinoids correlate to the presence of different terpene profiles related to different cultivars of *C. sativa* and the extraction method.

Collectively, these findings emphasize the potential of cannabinoids as immunomodulators, offering promise for future anti-inflammatory therapies. Research in this area has made strides in mitigating the severity of symptoms and slowing the progression of various inflammatory diseases [36,50,56,57,58], providing potential avenues for therapeutic advancements [39,43,53,59,60,61,62,63,64,65,66] (Figure 2A).

## 5. Cannabinoid Antimicrobial Properties

The potential antimicrobial properties of CBD were first reported as early as 1976 by van Klingeren and Ham, who found a minimum inhibitory concentration (MIC) between 1–5 µg/mL against gram-positive *Staphylococcus* and *Streptococcus* [67]. However, this potential largely remained unexplored until 2008, when Appendino et al. demonstrated its efficacy against six strains of methicillin-resistant *Staphylococcus aureus* (MRSA) with an MIC between 0.5 and 2 µg/mL [68].

Cannabis essential oils and cannabinoids have shown effectiveness against methicillin-resistant *S. aureus*, *Helicobacter pylori*, *Enterococcus faecalis*, and the ability to interfere with the quorum sensing cascade in *Vibrio harveyi* [68,69,70,71]. Moreover, cannabinoids have displayed potential antimicrobial properties against a wide range of bacterial species, both gram-positive and gram-negative, and various fungi [22,72,73]. The presence of terpenes in the profiles of these compounds seems to correlate with their effects [74].

Some studies have reported a MIC between 1 and 4 µg/mL (3.17–12.7 µM) against a diverse range of gram-positive bacteria, including MRSA, *Streptococcus pneumoniae*, *Enterococcus faecalis*, and anaerobic bacteria such as *Clostridium difficile* and *Cutibacterium acnes*. Notably, these MIC values did not significantly change even against highly resistant bacteria, including *S. aureus*, vancomycin-resistant enterococci, and hypervirulent *C. difficile* strain 027. However, CBD was less potent against certain beta-hemolytic *Streptococcus* species (*Streptococcus pyogenes* and *Streptococcus agalactiae*) with MIC values between 8 and 32 µg/mL (25.4–101.7 µM). Surprisingly, CBD showed excellent potential against some gram-negative bacteria, including *Neisseria gonorrhoeae*, *Neisseria meningitidis*, *Moraxella catarrhalis*, and *Legionella pneumophila*, with MIC values ranging from 0.25 to 2 µg/mL^−1^. CBD, however, was not effective against efflux pump-resistant strains of *Escherichia coli* or *Pseudomonas aeruginosa* (MIC >128 µg/mL^−1^), suggesting that some gram-negative bacteria capable of generating this resistance mechanism may not be sensitive to CBD [24].

CBG, on the other hand, has demonstrated remarkable efficacy against various gram-positive bacteria, particularly *S. aureus*. In comparison with traditional antibiotics such as norfloxacin and erythromycin, CBG exhibited a significantly lower MIC of 1 µg/mL and even outperformed CBD. Additionally, CBG displayed a lower MIC compared to tetracycline and oxacillin in at least one of the six strains [68]. In planktonic states, the MIC for *S. aureus* using CBG was 2 µg/mL (6.3 µM) and exhibited the highest anti-biofilm activity, inhibiting biofilm formation by 50% at a 0.5 µg/mL dose. Moreover, CBG demonstrated the ability to eradicate pre-formed biofilms of methicillin-resistant *S. aureus* USA300 at a concentration of 4 µg/mL (12.6 µM) [75,76].

Taken together, these findings underscore the promising antimicrobial potential of CBD and CBG, especially against drug-resistant bacterial strains, offering potential avenues for future antimicrobial therapies (Figure 2B).

## 6. Cannabinoid Potential in the Treatment of Periodontitis

### 6.1. Effects of Cannabinoids on the Oral Microbiota

In the context of periodontal health, the antibacterial and antifungal properties of cannabinoids concerning oral microbiota have been explored. Despite reports of promising results, there is still a long way to go; the existing scientific evidence regarding this topic is summarized below.

CBG has been found to reduce the expression of biofilms of *Streptococcus mutans*, inhibiting their formation at a minimum biofilm inhibitory concentration (MBIC) of 2.5 μg/mL and decreasing metabolic activity at higher concentrations (10 μg/mL). It also downregulates biofilm-related genes and quorum sensing-related genes [77]. Similarly, high doses of CBD, CBN, and THC suppressed the growth of *Porphyromonas gingivalis* and *Filifactor alocis*, but *Treponema denticola* was resistant to all tested doses. THC also had a prebiotic-like effect by preserving commensal bacteria. THC’s impact on the microbiota was particularly noteworthy [77].

CBD has also been evaluated for its antifungal effects on *Candida albicans*; while a minimum inhibitory concentration (MIC) was not identified, CBD displayed a dose-dependent disruptive effect on biofilm formation. Specifically, CBD inhibited biofilm formation by 37% after 24 h (12.5 µg/mL) and disrupted mature biofilms by up to 44% after 72 h (3.12 µg/mL). Also, higher concentrations reduced the metabolic activity of mature biofilms [78]. Similarly, Endocannabinoids AEA and AraS have been reported to inhibit the yeast–hyphal transition and prevent the adhesion of hyphae to epithelial cells in *C. albicans* [79].

In general, Cannabinoids seem to have shown more efficacy in reducing bacterial colony counts compared to established commercial synthetic oral care products. CBN and CBC were found to be particularly effective. Cannabinoid-based mouthwashes demonstrated bactericidal efficacy comparable to chlorhexidine (CHX) [80]. However, the studies reviewed had certain limitations, including sample size and a diverse range of participants with gingivitis and periodontitis, which makes it necessary to conduct randomized controlled trials focused on patients with periodontitis to evaluate the long-term effects of cannabinoids on the periodontal biofilm. Additionally, in vitro design and specific cell cultures were used in these studies, indicating the need for more research to elucidate the mechanisms of cannabinoid antimicrobial action and determine their effectiveness and applicability in controlling periodontal pathogens. Further studies are required to address these aspects comprehensively. 

### 6.2. Expression of Cannabinoid Receptors in the Periodontium

The expression of cannabinoid receptors in the periodontium has been studied in relation to the presence or absence of disease and the restorative states of the tissues. Cannabinoid receptors are expressed in gingival fibroblasts and periodontal ligament (PDL) cells [81,82]. Regarding the expression patterns in periodontitis, it has been found that both CB1 and CB2 are upregulated under pathological conditions [83,84]. This is consistent with previous studies that indicated the activation of immune cells in response to LPS or other pro-inflammatory stimuli upregulates the expression of these receptors in systemic conditions such as colon pathologies [56,85]. In this way, in periodontal tissues, Konermann et al. demonstrated that sterile inflammation increases the expression of CB1 and CB2 [82].

In a healthy periodontium, CB1 receptors are reported to be highly expressed in the PDL. These receptors are generally located in periodontal tissues, including the junctional epithelium, gingival connective tissue, PDL, and alveolar bone surface [86]. CB2 ligands are linked to anti-inflammatory functions related to the modulation of periodontal cell migration and adhesion through focal adhesion kinase (FAK) and mitogen-activated protein kinase (MAPK) systems [87]. 

**Figure 3:**
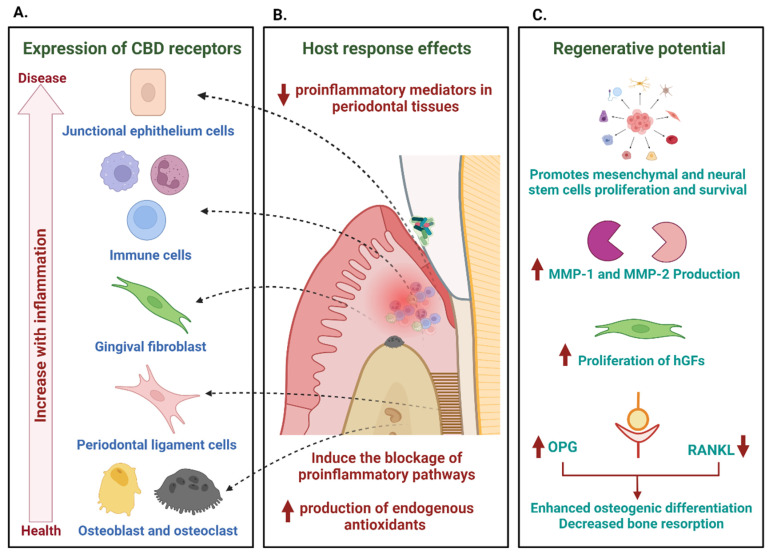
Potential effects of cannabinoids in the control of periodontal inflammation. The expression of CB1 and CB2 cannabinoid receptors in periodontal tissues (junctional epithelium, immune response cells, gingival and periodontal ligament fibroblasts, osteoblasts, and osteoclasts) and the increase in their expression in the presence of inflammation are a clear indication of a possible role in modulating the host response (**A**). Through their activation, cannabinoids mediate anti-inflammatory and regulatory responses, decreasing mediators of inflammation in periodontal tissues and inducing the blocking of pro-inflammatory pathways (**B**). The modulation of these responses goes hand in hand with a range of actions that promote the repair and regeneration of tissues, which include the promotion of the proliferation and survival of stem cells, the increase in the production of metalloproteinases, hand in hand with the proliferation of gingival fibroblasts (hGFs) and anabolic functions of bone metabolism mediated by their activity in the RANK–RANKL–Osteoprotegerin (OPG) system, which results in an increase in osteogenic differentiation and a decrease in bone resorption (**C**).

According to a histomorphometric study of healthy individuals, CB1 and CB2 receptors are expressed equally, with no difference between the epithelium and connective tissue. However, during an inflammatory process, whether in relapsing or non-relapsing patients, the expression of CB1 and CB2 receptors undergoes upregulation. Notably, the superficial layers, including the granular layer and the corneum stratum of the epithelium, exhibit significantly higher receptor levels, as does the connective tissue. In sites affected by inflammation and recurrent disease, cannabinoid receptor counts were significantly higher compared to non-recurrent and healthy sites [88] (Figure 3A).

The activation of cannabinoid receptors and their mechanism of action have become increasingly important in defining potential therapeutic targets. In periodontal tissue, there is an observed upregulation of CB1/CB2 expression localized in fibroblasts and macrophage-like cells within granulation tissue during the healing process [81]. 

### 6.3. Effects of Cannabinoids on Periodontal Inflammation Modulation

In vitro studies show that both endo and exo-cannabinoids reduce the inflammatory response in periodontal tissue by significantly reducing the production of pro-inflammatory mediators (Figure 3B). The evidence comes from some in vitro and in vivo experiments in animals mainly focused on the activation of factors and signal transcription pathways important in the activation of the production of pro-inflammatory factors. 

In vitro studies show that AEA reduces the LPS-induced inflammatory response of *P. gingivalis* by significantly decreasing the production of pro-inflammatory mediators [89]. Its immunosuppressive activities in periodontal cells have been associated with the blockade of NF-κB activation [83]. Therefore, AEA could be a promising regulator of an immune challenge since it maintains the physiological function of cells [84] and, in addition, effectively counteracts the positive regulation of inflammatory cytokines [90].

It is important to highlight that endocannabinoids AEA and 2-AG are detectable in gingival crevicular fluid, and their level appears to increase in individuals with periodontal disease [86]. 

As for exo-cannabinoids, CBD can bind to the peroxisome proliferator-activated receptor gamma (PPAR-γ) (whose expression expands in periodontal disease) [91], increasing its transcriptional activity and modulating the immune response and binding results in a decreased production of cyclooxygenase-2 (COX-2) and pro-inflammatory cytokines such as IL-1β, IL-6, and TNF-α. Additionally, the activation of PPAR-γ can regulate the expression of the transcription factor Nrf2, which is responsible for producing endogenous antioxidants in the periodontal environment [92]. 

THC has also been proposed as an anti-inflammatory drug in the gingiva using an HIV/SIV infection model in rhesus macaques. During chronic infection, CD4+ T cells and macrophages in the gingival lamina propria exhibit increased viral replication, leading to increased pro-inflammatory mediators (such as CXCL10 and MMP12). This, in turn, results in the activation of Indoleamine 2,3 dioxygenase 1 (IDO1), the downregulation of Dual oxidase 1 (DUOX1), salivary dysbiosis, and a reduced expression of gingival epithelial junction proteins like Desmocollin 3 (DSC3). The administration of D9-THC (delta-9-tetrahydrocannabinol) can maintain DUOX1 by blocking the upregulation of miR-125a-5p and decreasing IDO1 protein expression in the apical epithelium via a CB2-mediated mechanism. Low-dose cannabinoids could also inhibit the upregulation of interferon-γ-inducible protein 10 (CXCL10) and matrix metalloproteinase 12 (MMP12) [93].

In vitro treatment with cannabinoids in gingival cells [77] and mesenchymal stem cells [94] demonstrates their anti-inflammatory potential by altering the cytokine release profile and enhancing the release of IL-10 and TGF-β [95]. This supports the significance of this review in exploring whether cannabinoids’ physiological functions in periodontal healing also encompass direct actions on regeneration mechanisms. The integrity of periodontal tissues, which includes the health of the periodontium and the success of periodontal treatment, relies on its regenerative potential [96].

### 6.4. Regenerative Potential of Cannabinoids at the Periodontium

The endocannabinoid system (ECS) is known to be involved in spinal cord, colon, and liver repair [97,98,99]. However, research on the ECS in the context of the periodontium is limited at present. Nonetheless, the available findings attribute an essential role to cannabinoids and the endocannabinoid system in periodontal healing [81], particularly in regulating alveolar bone metabolism.

Qian et al. showed that the activation of CB2 by HU-308, a CB2-specific agonist, stimulates the expression of osteogenic genes in human periodontal ligament (hPDL) cells. These genes include the Runt-related transcription factor 2 (Runx2), the bone sialoprotein (BSP), osteopontin (OPN), alkaline phosphatase (ALP), osteocalcin (OC), and collagen I (COL I). This heightened expression leads to osteogenic differentiation, an increase in osteoprotegerin (OPG) [100], and a reduction in the expression of nuclear factor-kappa B ligand (RANKL). Furthermore, CBD diminishes bone resorption by inhibiting the expression of RANK/RANKL in the alveolar bone during experimentally induced periodontitis in rats.

Over a 30-day period, the rats received 5 mg/kg of CBD (2%) dissolved in a saline solution vs. a placebo. Rats in the CBD group exhibited lower expression of the NF-Kβ ligand RANK/RANKL and experienced less alveolar bone loss. The production of IL-1β and TNF-α was also reduced [101]. Since the ratio of OPG to RANKL may indicate the role of hPDL cells in bone resorption, the results suggest that CB2 activation could inhibit osteoclastic activity and enhance the osteogenic differentiation of hPDL cells [101].

Both osteoblasts and osteoclasts express CB2 [30]. Animal experiments have demonstrated that CB2-deficient mice exhibit significantly accelerated age-related bone loss, while CB2 activation mitigates ovariectomy-induced bone loss in mice by promoting bone formation and restraining bone resorption [102]. Notably, in an experimental model of periodontitis in rats, there was an increase in alveolar bone loss following the blockade of CB1 and CB2 receptors [103]. Additionally, in a study investigating the role and mechanism of CB1 in the osteo/dentinogenic differentiation of periodontal ligament stem cells, Yan et al. discovered that CB1 enhances the osteo/dentinogenic differentiation potential of PDL stem cells by upregulating key transcription factors, including Osterix (OSX), Distal-Less Homeobox 2 (DLX2), Distal-Less Homeobox 3 (DLX3), and Distal-Less Homeobox (DLX5), which are crucial during osteo/dentinogenic differentiation [104].

Recent studies propose that CB1 activation enhances the osteogenic differentiation potential and mitochondrial energy metabolism in human bone marrow mesenchymal stem cells (hBMSCs) through the c-Jun N-terminal kinase (JNK) signaling pathway and p38 mitogen-activated protein kinase (p38 MAPK). This activation also elevates the expression of nuclear respiratory factor 1 (Nrf1) and nuclear respiratory factor 2 (Nrf2), members of the Cap n Collar (CNC) transcription factor family. These findings indicate the potential role of CB1 in mesenchymal stem cells and periodontal regeneration, making it a promising target for bone regeneration [105]. In contrast, CB2 activation has been reported to reduce alveolar bone loss in rats with periodontal disease [106]. Additionally, THC in periodontal fibroblasts promotes adhesion and migration, primarily through CB2 activation [88].

It was also recently reported that stem cells derived from different dental tissues can differentiate into osteoblasts under various conditions using vitamin D3 and CBD. Low doses of CBD induce greater expression of bone proteins for stem cells derived from the apical papilla, and those from the dental follicle demonstrated a high mineralization capacity using CBD [107].

The study by Montreekachon et al. observed that even doses of cannabidiol up to 30 μM did not induce toxicity in human gingival fibroblasts (HGFs). Treatment with cannabidiol at concentrations of 3 or 10 μM significantly increased the mean percentage of cell proliferation (*p* < 0.001). This enhancement was consistent with the upregulation of cyclin D1 and Ki-67 expressions, along with increased percentages of HGFs in the S and G2/M phases [108].

Kozono et al. reported increased levels of AEA in the gingival crevicular fluid (GCF) after periodontal surgery in patients with periodontitis. After in vitro analysis, they observed that AEA significantly promoted the proliferation of the human gingival fibroblast [81], an increase previously reported in bone marrow lesions [98]. Also, the upregulation of CB1/CB2 expression localized to fibroblasts and macrophage-like cells in granulation tissue during healing has been observed. THC in periodontal fibroblasts promotes their adhesion and migration, which are primarily dependent on CB2 [87]. 

It has also been reported that CBD treatment of human gingival mesenchymal stem cells (hGMSCs) suppresses the levels of the Nod-like receptor family pyrin domain containing 3 (NALP3) inflammasome, caspase 1 (CASP1), and IL-18, changing the cellular phenotype and reducing the risk of inflammatory reactions [109]. 

Additional evidence supports the action of other components of the endocannabinoid system in the healing process. Rawal et al. reported that after 1 to 6 days of incubation of gingival fibroblasts with CBD alone, TGF-β and fibronectin production were increased. Also, the production of matrix metalloproteinase (MMP)-1 and MMP-2 increased with concentrations of 0.1–0.5 µM of CBD; with higher concentrations, these MMPs’ production decreased [95]. 

Among the cellular effects that enhance the impact of cannabis on regenerative events is the ability to improve the survival and differentiation of stem cells; treatment with CBD in combination with Morigin (a compound obtained from Moringa oleifera seeds) in mesenchymal stem cells of the periodontal ligament (hPDLSC) showed improved survival by inhibiting apoptosis, increasing the expression of anti-apoptotic genes (XIAP, HSPB1, HSP90AA1, and HSPA4) and decreasing the activation of NF-κB, caspase 8, caspase 9, caspase 10, caspase 6, and caspase 7 [110] (Figure 3C).

The expression of CB1 and CB2 cannabinoid receptors in periodontal tissues (junctional epithelium, immune response cells, gingival and periodontal ligament fibroblasts, osteoblasts, and osteoclasts) and the increase in their expression in the presence of inflammation are a clear indication of a possible role in modulating the host response (A). Through their activation, cannabinoids mediate anti-inflammatory and regulatory responses, decreasing mediators of inflammation in periodontal tissues and inducing the blocking of pro-inflammatory pathways (B). The modulation of these responses goes hand in hand with a range of actions that promote the repair and regeneration of tissues, which include the promotion of the proliferation and survival of stem cells, the increase in the production of metalloproteinases, hand in hand with the proliferation of gingival fibroblasts (hGFs) and anabolic functions of bone metabolism mediated by their activity in the RANK–RANKL–Osteoprotegerin (OPG) system, which results in an increase in osteogenic differentiation and a decrease in bone resorption (C). 

## 7. Conclusions

Examining the endocannabinoid system and the physiological impacts of cannabinoids on the periodontium has unveiled immunomodulatory and antibacterial properties, potentially contributing to the proper healing and regeneration of periodontal tissues. Although more evidence is required for precise conclusions, the current data support ongoing research into these agents as a viable option for therapeutic interventions in cases of periodontitis.

It is proposed to construct in vitro and in vivo models to assess the potential effectiveness of cannabinoids in preventing the formation of oral biofilms and disrupting established ones. Additionally, the development of carriers ensuring their timely release at the intended site, followed by clinical studies demonstrating efficacy in humans, is suggested. This includes considering cannabinoids as both an adjunct therapy to mechanical treatment and an agent with potential roles in the repair and regeneration of disease sequelae.

## Data Availability

Not applicable.
